# Relationship between satisfaction with mental health services, personal recovery and quality of life among service users with psychosis: a cross-sectional study

**DOI:** 10.1186/s12913-021-06409-0

**Published:** 2021-05-08

**Authors:** Regina Skar-Fröding, Hanne Kristin Clausen, Jūratė Šaltytė Benth, Torleif Ruud, Mike Slade, Kristin Sverdvik Heiervang

**Affiliations:** 1 R&D Department, Division of Mental Health Services, Akershus University Hospital, P.O. box 1000, 1478 Lørenskog, Norway; 2 Norwegian National Advisory Unit on Concurrent Substance Abuse and Mental Health Disorders and Mental Health Division, Innlandet Hospital Trust, Brumunddal, Norway; 3 Institute of Clinical Medicine, Campus Ahus, University of Oslo, Oslo, Norway; 4 Health Services Research Unit, Akershus University Hospital, Lørenskog, Norway; 5 School of Health Sciences, Institute of Mental Health, University of Nottingham, Nottingham, UK; 6 Centre for Medical Ethics, Faculty of Medicine, University of Oslo, Oslo, Norway

**Keywords:** Service satisfaction, Mental health service user, Personal recovery, Quality of life, Community treatment order

## Abstract

**Background:**

Mental health policy internationally emphasizes patient centredness and personal recovery. This study investigated the relationship between satisfaction with mental health services among service users with psychosis in Norway, and personal recovery, perceived support for personal recovery, and quality of life.

**Methods:**

Cross-sectional data were collected from 292 service users diagnosed with psychosis from 39 clinical sites across Norway. Satisfaction with services was assessed using the Client Satisfaction Questionnaire-8. A linear mixed model was estimated to explore the relationship between satisfaction with services and preselected covariates, and to control for confounding factors.

**Results:**

A large majority of participants (89%) reported moderate-to-high levels of satisfaction. Satisfaction with services was positively associated with perceived support for personal recovery, but not with personal recovery or quality of life. In addition, service users under a Community Treatment Order (CTO) were significantly less satisfied than those who were not.

**Conclusions:**

Satisfaction levels among service users were higher compared with similar, international studies. Those who feel supported in their personal recovery were more satisfied with the care they receive, which support the need for implementation of recovery-oriented practices for service users with psychosis. However, satisfaction with services was not related to service user-rated quality of life or level of personal recovery; thus, more follow-up studies are needed. The lower satisfaction of service users placed under CTOs shows the importance of targeted interventions to improve satisfaction with services among this group.

**Trial registration:**

NCT03271242, date of registration: 5 sept. 2017.

## Background

Satisfaction with services is widely regarded as an important process variable and quality indicator in mental health care [1, 2]. In general, satisfied service users are more adherent to treatment and benefit more from care [1], while those who are less satisfied have poorer treatment outcomes [3]. Satisfaction with services can be influenced by user characteristics and by treatment and services aspects [4]. Among socio-demographic characteristics the only feature consistently linked with satisfaction with care has been service user age, with higher age associated with higher satisfaction. Clinical characteristics and self-reported outcome measures have shown more substantial correlations. Higher symptom level (especially more depressive symptoms), personality disorder diagnosis, and lower self-reported quality of life have been found to be associated with less satisfaction with care [5]. Among service characteristics, only coercive treatment and a perceived negative therapeutic relationship have been consistently found to impact satisfaction with care [1, 3]. Coercion appear to have a key role in ratings of satisfaction [4]. Overall, when evaluating satisfaction with services, the identified confounders are age, legal status of treatment, and severity of illness or symptoms, particularly depressive symptoms [1].

Satisfaction with services is also an important quality indicator among services users with psychosis [5]. Among service users with psychosis, higher satisfaction rates have been associated with clinical outcome benefits, such as reduction in positive psychotic symptoms at follow-up [6], and lower satisfaction rates have been associated with more involuntary admissions, more severe psychopathology, and more unmet needs [7].

Satisfaction rates for service users with psychosis seem to differ between countries. A previous study of 654 Dutch service users with psychosis showed satisfaction rates with mental health services ranging among low (19.4%), intermediate (48.9%), and high (31.7%) [6]. A total of 125 Israeli service users with psychosis reported themselves as dissatisfied (16.8%), barely satisfied (45.6%), moderately satisfied (25.6%), or highly satisfied (12%) [8]. Among 130 Kuwaiti service users with schizophrenia, the dissatisfaction rate was 21.5% [9], consistent with rates from a multisite European study showing dissatisfaction ranging from 26 to 42.2% among service users with psychosis at five sites [7].

Although satisfaction with services has been consistently associated with self-reported outcomes such as quality of life among the general mental health population [1], among service users with psychosis the relation between satisfaction and self-reported outcome measures is inconclusive and underexplored. One study showed a significant association between dissatisfaction with care and lower self-reported quality of life [7], while another showed positive associations between satisfaction and quality of life at baseline but not at follow-up [6]. In addition, another study among people with schizophrenia found that treatment satisfaction was high even though life satisfaction was low [10]. In sum, while objective clinical benefits of high service satisfaction seem apparent among service users with psychosis, studies show varying associations between satisfaction with services and other self-reported outcomes, such as quality of life and life satisfaction.

Furthermore, no attempts have been made to examine satisfaction with services and its relationship with self-reported personal recovery, although some studies have investigated satisfaction with recovery-related topics such as shared decision-making [11] and empowerment [12]. As healthcare systems in developed countries evolve from a paternalistic to a patient-centred approach [13] concepts like quality of life and personal recovery have received increased attention. The personal recovery concept originates from the user movement [14], and focuses on prioritizing more personal and subjectively meaningful treatment goals [15]. It is often contrasted to clinical recovery, the definition traditionally used in mental health services, which focuses on symptom reduction and increased function [16]. While clinical recovery has traditionally been the primary goal in the treatment of people with psychosis, supporting and focusing on personal recovery has become a key aim in mental health services in many countries [17] and has had a considerable impact on health care policy. The World Health Organization’s Comprehensive Mental Health Action Plan 2013–2020 [18], promotes a recovery orientation in mental health systems, emphasizing that the central issue for mental health services is to expand the understanding and knowledge of promoting recovery. In Norway, recent central political guidelines from the Ministry of Health and Care Services (Helse- og omsorgsdepartementet), have placed emphasis on developing more patient-centred care [19], consistent with requests from user organizations [20]. In the Norwegian national guidelines on assessment and treatment of persons with psychoses (2013), a recovery approach is emphasized as a general principle for good practice [21], and recent years have seen an increase in implementation of development recovery-oriented practices in Norway such as Illness Management and Recovery (IMR) [22] ACT/FACT –teams [23] and Individual Placement and Support (IPS) [24]. A previous study showed that a great majority of Norwegian service users with psychosis reported that support for personal recovery were important for them (article in press).

To sum up, while satisfaction with services is associated with beneficial clinical outcomes among individuals with psychosis, studies investigating the relationship between satisfaction and quality of life or life satisfaction have generated inconsistent findings. Furthermore, no study to date has investigated the relationship between satisfaction with services and personal recovery. If personal recovery and patient centeredness are to be the focus of mental health services policy, then examining their relations to user satisfaction with services is necessary. This has important clinical implications for mental health services since satisfaction with services should impact these important aspects of the lives of those with psychosis, in addition to more traditional clinical outcomes like reduced hospitalization and symptoms.

The aims of this study were to examine the level of satisfaction with services among service users with psychosis across Norway, and to examine the relations between satisfaction with services and personal recovery, perceived support for personal recovery and quality of life. Based on existing findings on quality of life and the importance of the therapeutic relationship for satisfaction with services, we hypothesized that higher quality of life and more perceived support for personal recovery would be positively associated with higher satisfaction with services. We also expected that service users who are highly satisfied with services would report higher levels of personal recovery. Finally, we hypothesized that users in a Community Treatment Order (CTO) would be less satisfied with their care.

## Methods

### Design

This is a cross-sectional study, analyzing baseline data from the Norwegian research project A Pairwise Randomized Study on Implementation of Guidelines and Evidence-based Treatments of Psychoses (ClinicalTrials NCT03271242). The study was approved by the Regional Committee for Medical and Health Research Ethics (REK Sørøst B 2015/2169), and followed the principles of the Declaration of Helsinki.

### Setting and sample

A total of 325 mental health service users from six health authorities across Norway, including three university hospitals, were recruited. Thirty-nine clinical units and hospital departments with outpatient clinics, day units, mobile teams, and inpatient wards participated. Further details about the participating units are available in the study protocol (ClinicalTrials NCT03271242). Inclusion criteria were: mental health service user diagnosed with psychosis (ICD-10 F20–29) (World Health Organization, 1992), and aged 16 years or older. The only exclusion criterion was being unable to understand and answer the questionnaires in Norwegian. Thirty-three service users with missing data were excluded, reducing the final study sample to *N* = 292.

### Measures

#### Outcome measure

The Client Satisfaction Questionnaire-8 (CSQ-8) (Table 1) [25] 8 is an eight-item questionnaire used to measure patient’s global satisfaction with services, which has shown good psychometric properties . The CSQ-8 measures general satisfaction on eight scaled items from 1 (= poor) to, 4 (= excellent) resulting in a total score range of 8–32. Level of satisfaction is classified as low [8–20], intermediate [21–25], or high [26–30] . Psychometric evaluation of CSQ-8 in the current sample showed high scale reliability (Cronbach’s alpha 0.91).

**Table 1:** Items of the Client Satisfaction Questionnaire (CSQ-8) (Range 8–32)

1. How would you rate the quality of service received?	
2. Did you get the kind of service that you wanted?	
3. To what extent has our program met your needs?	
4. If a friend were in need of similar help, would you recommend our program to him or her?	
5. How satisfied are you with the amount of help you have received?	
6. Have the services you received helped you to deal more effectively with your problems?	
7. In an overall, general sense, how satisfied are you with the service you have received?	
8. If you were to seek help again, would you come back to our program?	

#### Covariates

The Questionnaire about the Process of Recovery (QPR) [27] was used to examine personal recovery level. The QPR is a 15-item self-report measure of recovery developed through collaboration between clinicians and service user researchers, which has shown adequate psychometric properties [28]. Items are rated on a five-point Likert scale from 0 (Disagree strongly) to 4 (Agree strongly). Total sum score ranges from 0 (low recovery) to 60 (high recovery). Psychometric evaluation of QPR in the current sample showed a one factor solution with high scale reliability (Cronbach’s alpha 0.91).

Perceived support for personal recovery was examined using the 20-item support subscale from the *INSPIRE* measure of staff support of personal recovery [29]. Each service user-rated subscale item is first rated on whether it is important for the participant’s recovery (e.g. “An important part of my recovery is … feeling hopeful about my future”, (Yes/No). If yes, the participant rates the support they receive from their health service provider for this item (“I feel supported by my worker with this”) on a five-point Likert scale from 0 (Not at all) to 4 (Very much). A total support score is calculated for each participant as described in the INSPIRE scoring instruction guide (http://www.researchintorecovery.com/INSPIRE#s12) and ranges from 0 (low support) to 100 (high support). Psychometric evaluation of the INSPIRE in the current sample showed a one factor solution, with a good internal consistency (Omega coefficient 0.96) [30].

Quality of life was assessed using a single item (Item 1, Life as a whole) from the *Manchester Short Assessment of Quality of Life (MANSA*) [31]: “*How satisfied are you with your life as a whole?”* which was rated on a seven-point scale from 1 (Couldn’t be worse) to 7 (Couldn’t be better). The variable was named Quality of life. MANSA item 1 (Life as a whole) has been shown to correlate strongly (Pearson correlation coefficient .832, *p* < .001) with item 1 (Life as a whole) in the Lancashire Quality of Life Profile (LQoLP) [32].

#### Confounders

User and service characteristics that have been considered as potential confounders in studies on satisfaction with services, such as illness severity, depressive symptoms, age, and legal status of treatment [1], were included.

Illness severity was assessed using the Global Assessment of Functioning Scale (GAF) [33]. Level of functioning and severity of service users’ symptoms are rated by clinicians on a scale (1–100), with lower scores indicating more severe symptoms and lower levels of functioning. The split version of the scale used in this study has symptom (GAF-S) and function (GAF-F) subscales [34].

Depression was assessed using the ‘depression/functioning’ domain of the Behavior and Symptom Identification Scale (BASIS-24). BASIS-24 is a brief service user self-report measure of psychopathology and functioning, which was developed to assess mental health treatment outcomes. This 24-item scale assesses six symptom and functioning domains: Depression/functioning, Interpersonal relationships, Self-harm, Emotional lability, Psychosis, and Substance abuse. BASIS-24 has shown good validity and reliability for assessing mental health status and functioning from the perspective of service users [35, 36]. Scores were calculated as described in the BASIS-24 instruction guide [37], providing a score between 0 and 4 with higher scores indicating more severe problems.

Information on whether participants were on a CTO (Yes/No) at the time of participation in the study, gender, and age were also included as confounders.

#### Procedure

Clinicians at the participating mental health units recruited eligible service users who were in contact with the clinic during the study period, and newly referred service users assessed to have psychosis. Clinicians performed clinical ratings and questionnaires were administered to service users by the secretary or other clinic personnel. Service users were either provided with a place to sit in the clinic to complete the questionnaires, or took them home. When finished, the questionnaire was sealed in an envelope, and returned to the clinic. The recruitment period lasted from June 2016 until March 2017, and only participants who gave written informed consent were included.

#### Analysis

As participants came from different clinical sites nested within different health authorities, a hierarchical structure may have been present in these data. Intra-class correlation coefficient (ICC), representing a proportion of total variance that is between the sites and/or health authorities, was used to assess possible cluster effects. Because of noticeable cluster effect within clinical sites (ICC = 12.5%) and health authorities (ICC = 4.1%), unadjusted and adjusted linear mixed models with random effects for clinical sites nested within health authorities were estimated to assess the association between service satisfaction (CSQ-8) and three covariates (QPR, INSPIRE, MANSA) controlled for confounders (GAF-symptoms, GAF-Function, Depression/functioning, CTO, Age, and Gender). Multicollinearity was assessed by inspecting correlations among covariates, but no multicollinearity issues were found. Standard residual diagnostics was performed.

As an exploratory analysis, the interactions between being on a CTO and quality of life (MANSA) and personal recovery (QPR) were entered into the model, to determine whether the CTO-variable moderated their relation with CSQ-8. All tests were two-tailed, and results with *p*-values below 0.05 were considered statistically significant.

Imputation of missing values on the GAF (*n* = 34), MANSA (*n* = 6), QPR (*n* = 21) and CSQ-8 (*n* = 6) were performed by generating the empirical distributions for each variable and drawing a random number from that distribution to replace the missing value. The process was repeated until all missing values were imputed. Missing values on demographic variables were not imputed.

## Results

### Clinical and sociodemographic characteristics of participants

The mean age of the 292 participants was 40 years (Standard deviation (SD) = 12.7). Forty-two per cent were female. The majority of the participants (*n* = 277, 88%) defined themselves as Norwegian, and 15 (12%) were from other ethnic backgrounds. Fifty-three per cent had a diagnosis of schizophrenia and 13% were under CTO. Further details on sociodemographic and clinical characteristics of the 292 participants are shown in Table 2.

**Table 2:** Participants (*N* = 292) sociodemographic and clinical characteristics

Characteristics	
Gender N (%)	
Female	122 (42)
Ethnicity N (%)	
Norwegian	255 (88)
Other	34 (12)
Age Mean (SD)	40 (12.7)
Diagnosis N (%)	
Schizophrenia	145 (53)
Schizoaffective disorder	54 (20)
Other	74 (27)
GAF symptom^a^ Mean (SD)	53 (13)
GAF function^b^ Mean (SD)	51 (11.3)
Community treatment order N (%)	
Yes	40 (14)
Depression (BASIS-24)^c^ Mean (SD)	1.3 (0.92)
Personal recovery (QPR)^d^ Mean (SD)	41 (10.2)
Perceived support (INSPIRE)^e^ Mean (SD)	66 (17.6)
Quality of life (MANSA)^f^ Mean (SD	41 (10.2)
Satisfaction with services (CSQ-8)^g^ Mean (SD)	26 (4.7)

^a^Range from 0 to 100, higher scores indicate less severity

^b^Range from 0 to 100, higher scores indicate higher function

^c^Range from 0 to 4, higher scores indicate more severe symptoms

^d^Range from 0 to 60, higher scores indicate higher level of personal recovery

^e^Range from 0 to 100, higher scores indicate more perceived support

^f^Range from 1 to 7, higher scores indicate higher quality of life

^g^Range from 8 to 32, higher scores indicate higher satisfaction

### Service satisfaction amongst Norwegian service users with psychosis (*N* = 292)

The mean (SD) CSQ-8 score was 25 (4.7), indicating an average of intermediate satisfaction. The distribution was 30 (10%) reported low satisfaction, 141 (49%) reported intermediate satisfaction and 121 (41%) reported high satisfaction.

### Associations between satisfaction with services and personal recovery, perceived support for personal recovery and quality of life

Table 3 shows the results of the linear mixed model analysis performed to assess the associations between satisfaction with services (CSQ-8) and covariates. In the adjusted model, higher perceived support for personal recovery (INSPIRE) was associated with higher service satisfaction. Neither personal recovery (QPR) nor Quality of life (MANSA) showed significant associations with service satisfaction. There was an association with one confounder: service users on a CTO were significantly less satisfied than those not on a CTO.

**Table 3:** Linear mixed model results for associations between satisfaction with services (CSQ-8) and quality of life (MANSA), perceived support for personal recovery (INSPIRE), and personal recovery (QPR)

Covariates	Bivariate models			Multiple models		
	Regression coefficient	95% CI	*p*	Regression coefficient	95% CI	*p*
Personal recovery (QPR)^a^	0.12	0.07; 0.17	<.001	0.03	−0.03; 0.09	.354
Perceived support (INSPIRE)^b^	0.13	0.10; 0.15	<.001	0.11	0.08; 0.14	<.001
Quality of life (MANSA)^c^	0.56	0.18; 0.94	.004	0.07	−0.34; 0.47	.741
GAF-Symptom^d^	0.05	0.01; 0.10	.012	0.02	−0.03; 0.07	.440
GAF-Function^e^	0.05	0.005; 0.10	.031	−0.001	−0.06; 0.06	.962
Depression/functioning (BASIS-24)^f^	- 0.97	- 1.55; −0.40	.001	- 0.24	−0.88; 0.40	.460
CTO, yes	- 2.45	−3.96; −0.96	.002	−2.20	−3.57; −0.81	.002
Age	- 0.001	−0.05; 0.04	.692	−0.02	−0.06; 0.02	.263
Gender, female	1.45	0.38; 2.52	.008	0.98	−0.007; 1.96	.052

^a^Range from 0 to 60, higher scores indicate higher level of personal recovery

^b^Range from 0 to 100, higher scores indicate more perceived support

^c^Range from 1 to 7, higher scores indicate higher quality of life

^d^Range from 0 to 100, higher scores indicate less severity

^e^Range from 0 to 100, higher scores indicate higher function

^f^Range from 0 to 4, higher scores indicate more severe symptoms

The interactions between CTO status and quality of life (MANSA) and personal recovery (QPR) were not significant, and therefore not included in the regression models. Hence, CTO status did not account for the absence of association between CSQ-8 and either MANSA or QPR.

## Discussion

This cross-sectional study assessed the level of satisfaction with services, and its associations with self-reported outcome measures, among 292 service users with psychosis. Most (89%) of the service users rated their satisfaction as intermediate to high. Satisfaction with services was positively associated with perceived support for personal recovery, while personal recovery and quality of life were not significantly associated with service satisfaction. In addition, service users in a CTO were significantly less satisfied than those who were not.

Overall, our results showed higher satisfaction rates than those reported in similar international studies. Compared with the Dutch study [6], which also used the CSQ-8, covering 654 service users with psychosis, our results are somewhat more positive. However, despite somewhat similar samples and recruiting methods, their sample included many users experiencing their first episode of psychosis, and hence their sample were younger, which is often associated with less satisfaction. Results in our study, however, did not show age to be significantly related to satisfaction level. Compared with the studies form Israel [8], Kuwait [9] and the multi-site European study [7] our satisfaction scores seemed markedly higher. However, these other studies used different satisfaction scales, making adequate comparisons difficult.

Among European countries, Norway spends the largest share of its total health budget on mental health and has a significant element of tax financing for these services. Most health services are in the public sector, and deductibles are low [38]. In addition, significant efforts have been made to improve mental health care by increasing resources, and by making mental health care policy more patient centered and more highly prioritized [39]. However, our study shows that there is room for improvements, given that 11% of our participants reported low satisfaction rates. In particular, the low satisfaction scores among service users in a CTO adds further evidence for the importance of interventions aimed at improving satisfaction with services within this group.

As anticipated, our results show that service user who experience higher perceived support for personal recovery from their health care provider are more satisfied with the health care that they receive, and that those on a CTO are less satisfied. These findings are consistent with research concluding that among service characteristics, only coercive treatment and a perceived negative therapeutic relationship consistently impact satisfaction with care [1, 3]. This tells us that service-related factors important for the general mental health population are also relevant to service users with psychosis.

Support for personal recovery among service users with psychosis is associated with higher satisfaction, which is clinically important. For mental health clinicians, this means having an increased focus on recovery aspects such as the personal goals of the individual service user and finding out what matters most to them in their lives. Furthermore to strongly emphasize hope and empowerment when providing care for service users with psychosis. This finding also indicates that it is important to support service users with psychosis in their personal recovery, which calls for increased implementation of recovery-oriented practices. The question for mental health care services on how to target and improve quality of life and personal recovery of service users with psychosis is of great importance. Our previous study did show that recovery-oriented treatment (i.e., Illness Management and Recovery), is associated with higher perceived support for personal recovery (article in press), and another study has shown that service users enrolled in Assertive Community Treatment (ACT) programs are highly satisfied with the care they receive, including those being on a CTO [40]. Other interventions with a significant evidence base to support recovery include 19 randomised controlled trials of peer support work [41], 27 randomised controlled trials of the Individual Placement and Support approach to employment [42] and the development of education initiative called Recovery Colleges in 22 countries [43].

We did not find that satisfaction with services was related to quality of life or actual level of personal recovery. Perhaps this reflects that the perceived support for personal recovery more directly measures the service users’ experience with the mental health services, while actual level of personal recovery measures more broadly the general life situation of the person. Personal recovery and quality of life are concepts associated to many aspects in the life and community of the person. This is also why recovery-oriented interventions aimed at social inclusion such as Individual Placement and Support (IPS) [42] and Recovery colleges [43] has been developed and implemented.

However, as this is a cross-sectional study we do not have information on whether these concepts are related to satisfaction with services over time. Studies on the relations between satisfaction with services and self-reported outcomes among service users with psychosis are scarce; thus more follow-up studies measuring change over time are needed. These will be especially important since patient centeredness and personal recovery are the growing policy foci for mental health services. Such studies should also include clinical outcomes, to better explain how these three constructs interact. A mixed-methods study involving qualitative exploration of the experience of recovery support could also help develop an understanding of this process and inform the future development of more targeted interventions.

### Strengths and limitations

One of the major strengths of this study is that we adjusted scores for common confounders. This minimized the risk of positive and negative ratings being incorrectly attributed to service satisfaction when they actually reflect general tendencies of certain service users with specific characteristics (e.g., depressive symptoms), which can serve as a “mood-dominated” general tendency toward more positive or negative appraisals of one’s situation across various self-reported constructs [44].

One limitation is the common rater effect, a known potential bias when including several measures from the same respondent. However, our selected covariates showed only low-to-moderate correlations, speaking against such bias.

Selection bias was another potential risk. Although the 39 participating clinical sites are considered representative of psychosis treatment in the Norwegian mental health care system, participants were not randomly selected; thus, their satisfaction levels may not accurately represent the population with psychosis in Norway. In addition, although the clinicians that recruited the participants were instructed to recruit/ask all eligible service users, we do not have information of actual numbers of participants that were asked to participate. Hence, this sample might be a convenience sample which limits the generalizability of the findings and could explain the high satisfaction levels. Further, although CSQ-8 is among the most widely regarded scales for measuring service satisfaction, it does not cover all aspects of care. The scale also consist of questions of which some might be considered unsuitable to those on a CTO. Finally, as with all cross-sectional studies, it is not possible to draw any conclusions about causality.

## Conclusion

Satisfaction levels among service users were higher compared with similar, international studies. Service users who feel supported in their personal recovery were more satisfied with the care they receive, which calls for increased implementation of recovery-oriented practices for service users with psychosis. However, satisfaction with services was unrelated to quality of life or level of personal recovery. The low satisfaction reported by service users on a CTO emphasizes the importance of targeted interventions to improve satisfaction with services among this group.

## Data Availability

The datasets used and/or analysed during the current study are available from the corresponding author on reasonable request.

## Abbreviations

CTO: Community Treatment Order
IMR: Illness Management and Recovery
FACT: Flexible Assertive Community Treatment
IPS: Individual Placement and Support
ACT: Assertive Community Treatment
